# Amebic Liver Abscess in a Patient with Polycystic Liver Disease: A Case Report

**DOI:** 10.70352/scrj.cr.25-0488

**Published:** 2025-11-28

**Authors:** Mamoru Matano, Takanobu Hara, Hajime Matsushima, Akihiko Soyama, Ayaka Kinoshita, Takashi Hamada, Hajime Imamura, Tomohiko Adachi, Koichi Izumikawa, Takeshi Tanaka, Susumu Eguchi

**Affiliations:** 1 Department of Surgery, Nagasaki University Graduate School of Biomedical Sciences, Nagasaki, Nagasaki, Japan; 2 Infection Control and Education Center, Department of Infectious Diseases, Nagasaki University Hospital, Nagasaki, Nagasaki, Japan

**Keywords:** polycystic liver disease, liver abscess, amebiasis, *Entamoeba histolytica*

## Abstract

**INTRODUCTION:**

Amebic liver abscess is a parasitic infection caused by *Entamoeba histolytica*, which reaches the liver via the portal circulation after invading the colon. While it typically presents with fever, right upper quadrant pain, and elevated inflammatory markers, its clinical and radiologic features can resemble those of bacterial liver abscesses. In patients with structural liver abnormalities, such as polycystic liver disease, symptoms may be atypical and delay diagnosis.

**CASE PRESENTATION:**

We report the case of a 75-year-old woman with autosomal dominant polycystic kidney disease and multiple liver cysts, who presented with abdominal distension and gastrointestinal symptoms. MRI revealed multiple liver cysts, including a large lesion with heterogeneous signal intensity, initially suspected to represent intracystic hemorrhage. The patient underwent open cyst fenestration and deroofing. Postoperatively, she developed persistent ascitic fluid drainage and acute kidney injury requiring dialysis. Despite elevated inflammatory markers, she remained afebrile, and bacterial cultures from blood and ascitic fluid were negative. Histopathological analysis of the resected cyst wall revealed numerous trophozoites consistent with *E. histolytica*, and subsequent stool and ascitic fluid antigen tests confirmed the diagnosis. The patient was treated with a 16-day course of metronidazole followed by 7 days of paromomycin, resulting in clinical improvement and eventual discharge.

**CONCLUSIONS:**

For high-risk patients with complex hepatic cysts of uncertain cause, begin with percutaneous aspiration and targeted fluid analyses—including testing for *E. histolytica*—before invasive surgery; this lowers operative risk and aids detection of amebic liver abscess in atypical or culture-negative presentations.

## Abbreviations

ADPKD
: autosomal dominant polycystic kidney disease
ADPLD
: autosomal dominant polycystic liver disease
ALA
: amebic liver abscess
CRP
: C-reactive protein
HD
: hemodialysis
PCR
: polymerase chain reaction
PLA
: pyogenic liver abscess
PLD
: polycystic liver disease

## INTRODUCTION

Amebiasis is an infectious disease caused by the oral transmission of *Entamoeba histolytica*, which typically results in ulcerative lesions in the colon. In some cases, the parasite disseminates to extraintestinal organs, most commonly the liver, forming abscesses. ALAs usually present with symptoms such as fever, cough, and pain in the right upper quadrant or epigastrium within 2–4 weeks of onset. Gastrointestinal symptoms may also be present, and hepatomegaly with tenderness is a characteristic clinical finding.^1)^ While imaging modalities can readily detect hepatic lesions, there are no specific radiological features unique to ALA, making it difficult to distinguish them from PLAs.^2)^

PLD is a rare hereditary disorder characterized by mutations in genes that encode proteins involved in epithelial cell proliferation and fluid transport within the liver. PLD is classified into 2 major types: ADPKD and ADPLD.^3)^ Although patients are typically asymptomatic, those with significant hepatomegaly may experience abdominal pain or distension due to compression of adjacent organs, leading to impaired quality of life.^4,5)^ In symptomatic cases, treatment options include surgical interventions such as cyst fenestration, hepatic resection, or liver transplantation, along with interventional radiological procedures and pharmacotherapy aimed at reducing liver volume.^3,6)^ Complications of PLD include hemorrhage, rupture, and infection. Among these, infection is thought to result from bacterial translocation from the gastrointestinal tract. Although rare—occurring in approximately 1% of cases—infection can be diagnosed based on elevated inflammatory markers, localized pain, and positive blood cultures.^4)^

Herein, we report a rare case of ALA incidentally discovered during fenestration surgery for PLD.

## CASE PRESENTATION

The patient was a 75-year-old woman under urological care for ADPKD. She also had coexisting PLD and was referred to our department for further management due to symptoms suggestive of abdominal compression, including gastroesophageal reflux and urinary frequency. On physical examination, marked abdominal distension was observed, and the liver was palpably enlarged from the right upper quadrant to the flank. Laboratory tests revealed a mildly elevated CRP level of 2.59 mg/dL, while the white blood cell count was 6000/μL, within the normal range. Renal function was impaired due to ADPKD, with a blood urea nitrogen of 39 mg/dL and serum creatinine of 2.79 mg/dL. Liver function was preserved, with albumin 3.5 g/dL, aspartate aminotransferase 41 U/L, alanine aminotransferase 33 U/L, total bilirubin 0.5 mg/dL, and prothrombin time at 78%. There was no evidence of ascites. The tumor marker carbohydrate antigen 19-9 was elevated to 180 U/mL.

**Fig. 1:**
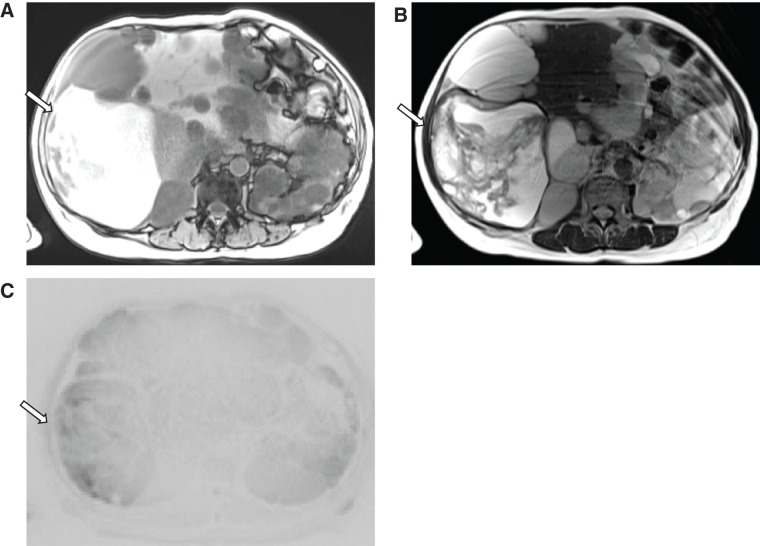
Preoperative MRI images. Abdominal MRI revealed multiple hepatic cysts, the largest measuring 15 cm in diameter (arrow). The largest cyst showed heterogeneous high signal intensity on T1-weighted imaging (**A**), T2-weighted imaging (**B**), and diffusion-weighted imaging (**C**).

Abdominal MRI revealed numerous cysts of varying sizes throughout the liver, with the hepatic parenchyma almost entirely replaced by cystic structures. The largest cyst measured approximately 15 cm in diameter and exhibited high signal intensity on both T1- and T2-weighted images, containing areas of heterogeneous low signal, suggestive of intracystic hemorrhage (**Fig. 1**). Due to the extensive hepatomegaly and renomegaly caused by the multiple cysts, there was insufficient space within the abdominal cavity, and thus, open surgical cyst fenestration and deroofing of the dominant cyst were planned.

**Fig. 2:**
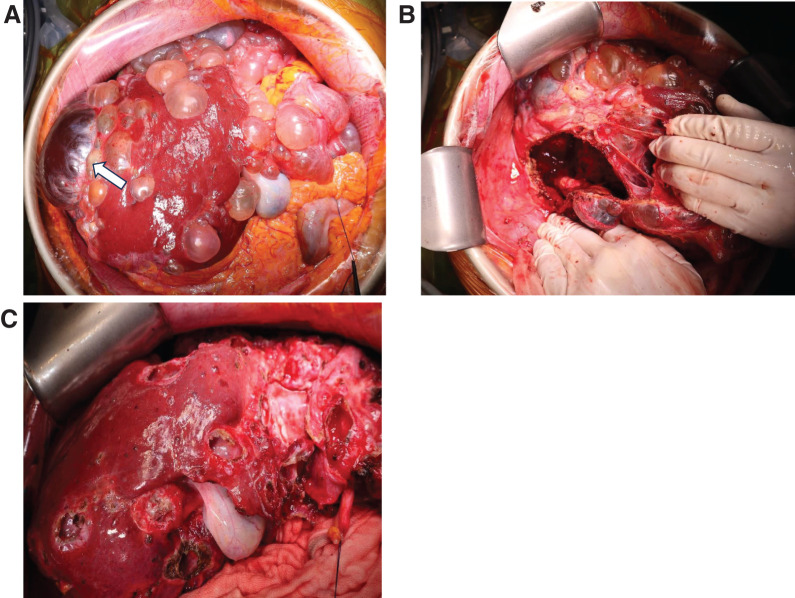
Intraoperative findings of open surgical liver cyst fenestration and deroofing. The largest cyst (arrow) had a thickened wall and contained brownish fluid (**A**). After fenestration of the largest cyst (**B**). After fenestration (deroofing) of multiple cysts (**C**).

Upon laparotomy, the largest cyst was identified in the right lateral segment of the markedly enlarged liver. The cyst wall appeared whitish, thickened, and degenerated, with findings suggestive of inflammation. The cyst contained brownish fluid, which was aspirated and removed, followed by deroofing of the cyst wall. To achieve durable decompression and symptom relief, we additionally performed targeted fenestration/deroofing of selected large or tense cysts that clearly contributed to hepatomegaly and mass effect. Although several cyst walls appeared whitish and thickened, there were no macroscopic signs of frank infection, and the operative strategy remained decompression rather than infection control. Serous, thin-walled cysts were then fenestrated in a stepwise manner to reduce overall cyst burden and the likelihood of early reaccumulation, resulting in a total of 4424 mL of cystic fluid being aspirated before concluding the procedure (**Fig. 2**). Cyst-fluid CA19-9 was not measured because the preoperative working diagnosis favored intracystic hemorrhage without suspicion of neoplasia. The operative time was 6 hours and 2 minutes, with minimal blood loss. Histopathological examination of the cyst wall revealed significant thickening, with the cyst lumen containing necrotic tissue and blood clots. The culture results of the cyst puncture fluid collected during surgery were negative.

**Fig. 3:**
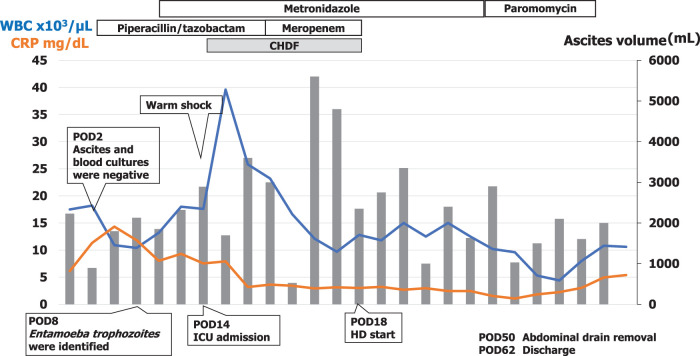
Postoperative clinical course. Bars show daily drainage volume from the abdominal drain. The fluid was lightly blood-tinged immediately after surgery and subsequently yellow serous. Timeline after open cyst fenestration/deroofing showing initiation of HD on POD 2, empiric tazobactam/piperacillin from POD 2, start of metronidazole on POD 9 followed by paromomycin, confirmation of *Entamoeba histolytica* antigen clearance in stool and ascitic fluid, drain removal on POD 50, and discharge on POD 64. CHDF, continuous hemodiafiltration; HD, hemodialysis

Following surgery, the patient developed ascitic drainage exceeding 2 L per day. The pre-existing chronic kidney disease acutely worsened, resulting in acute kidney injury on chronic kidney disease and initiation of HD on POD 2. Laboratory data showed an elevated white blood cell count of 18000/μL and a CRP level of 14.3 mg/dL, indicating an inflammatory response. However, the patient remained afebrile, and both ascitic fluid and blood cultures were negative. Suspecting intra-abdominal infection secondary to the drainage of infected cysts, empirical therapy with tazobactam/piperacillin was initiated on POD 2 (**Fig. 3**).

**Fig. 4:**
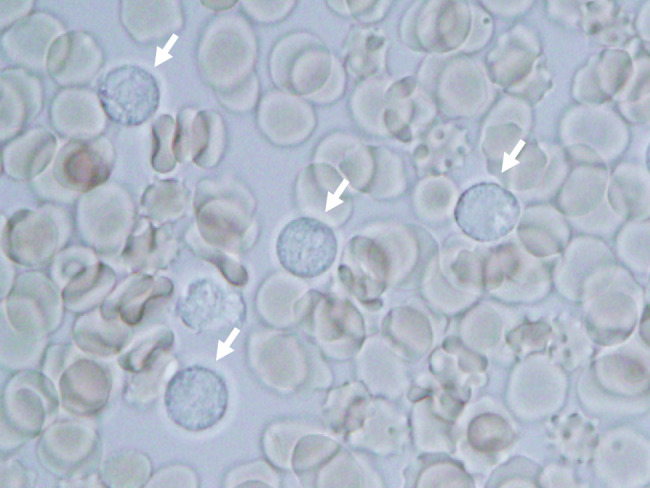
Subsequent smear examination of the ascitic fluid. Phagocytosed leukocytes with *Entamoeba* trophozoites were identified (arrows).

On POD 8, histopathological examination of the resected cyst wall revealed numerous round cystic organisms with phagocytosed leukocytes, consistent with *Entamoeba* trophozoites. Subsequent smear examination of the ascitic fluid also identified *Entamoeba* cysts (**Fig. 4**). Colonoscopy revealed no definitive ulcerations in the intestinal lumen, with only nonspecific erosions observed. However, a stool smear test was positive for *E. histolytica* antigen. Based on these findings, a diagnosis of ALA associated with multiple hepatic cysts and secondary peritonitis was established. The patient required temporary intensive care due to sepsis associated with peritonitis. Metronidazole was administered for 16 days starting on POD 9, followed by a 7-day course of paromomycin. Clearance of *Entamoeba* antigen was confirmed in both stool and ascitic fluid. After initiation of anti-amebic therapy, the daily drain output progressively decreased, and the fluid appearance changed from lightly blood-tinged in the immediate postoperative period to yellow, serous, without purulence. The abdominal drain was removed on POD 50. After creation of a vascular access shunt for HD and completion of rehabilitation, the patient was discharged on POD 64 (**Fig. 3**).

## DISCUSSION

ALA is a condition in which *E. histolytica*, following fecal–oral transmission and intestinal invasion, migrates to the liver via the portal circulation and forms abscesses within the hepatic parenchyma. While the majority of infections are asymptomatic or remain confined to the colon, approximately 1% of cases develop extraintestinal manifestations, most commonly involving the liver.^1,7)^ Notably, the absence of ulcerative lesions on colonoscopy does not exclude ALA: gastrointestinal manifestations are absent in many cases, and endoscopic findings may be normal or show only nonspecific erosions.^1,2,8)^ Our patient’s nonulcerative colonoscopy alongside positive *E. histolytica* antigen tests in stool and ascitic fluid is therefore consistent with the recognized spectrum of disease. Compared with PLA, ALA tends to occur more frequently in males aged 30–50 years, often without a history of hepatobiliary disease, and is typically associated with residence in or travel to endemic regions.^2,9)^ On imaging studies, both ALA and PLA present as similar space-occupying lesions, making parasitological or histopathological analysis essential for definitive diagnosis. A report evaluating the diagnostic utility of real-time PCR in ALA patients demonstrated sensitivities of 49% for blood samples, 77% for urine, and 69% for saliva, underscoring the potential role of molecular diagnostics in clinical practice.^10)^

Pharmacological therapy is the first-line treatment for ALA, with metronidazole—a member of the 5-nitroimidazole class—being the most commonly used and effective agent. Metronidazole targets the trophozoite form of *E. histolytica* within tissues; however, it has no effect on intraluminal cysts. Therefore, to prevent recurrence and transmission, a luminal agent such as paromomycin or, alternatively, diloxanide furoate should be administered in combination. It is important to note that paromomycin frequently causes diarrhea, which may confound the assessment of a patient’s response to treatment. For this reason, the simultaneous administration of metronidazole and paromomycin is generally not recommended.^1,11)^ Abscesses smaller than 5 cm can usually be managed with pharmacotherapy alone. However, in cases where the abscess is larger than 10 cm or where there is no clinical response to medication within 5–7 days, percutaneous or surgical drainage should be considered in conjunction with metronidazole therapy.^12)^

Our case represents an extremely rare instance of ALA diagnosed incidentally in the postoperative course following fenestration surgery for multiple hepatic cysts associated with ADPKD. At initial presentation, the patient was afebrile, demonstrated only mild inflammatory markers, and exhibited preserved liver function. Although some cysts showed heterogeneous changes on preoperative imaging, intracystic hemorrhage was initially suspected. Given the hepatomegaly and gastrointestinal compression caused by PLD, ALA was not considered as a differential diagnosis based on abdominal pain or gastrointestinal symptoms. The diagnosis was ultimately established postoperatively when histopathological examination revealed *E. histolytica* trophozoites phagocytosing leukocytes within the cyst wall, and antigen tests for *E. histolytica* were positive in both stool and ascitic fluid samples. A stool antigen test was also performed on the patient’s cohabitating husband, which returned negative. Furthermore, neither the patient nor her husband had a history of travel abroad, and they resided in a non-endemic area, making it impossible to identify a definitive route of infection in this case. In retrospect, given the patient’s high surgical risk and the difficulty of differentiating hemorrhage from infection on imaging, an initial percutaneous aspiration with comprehensive fluid studies (including *E. histolytica* testing) should have been considered. A preoperative diagnosis might have permitted medical management and avoided the morbidity of open surgery.

Amebic peritonitis is most commonly attributed to rupture of an ALA rather than to iatrogenic causes. Prior series describe overall rupture rates ranging roughly from 6% to 40%, pleuropulmonary complications in a notable subset, and intraperitoneal rupture patterns classified as “contained” (localized subphrenic/subhepatic collections) versus “free” rupture causing generalized peritonitis.^2,13)^ Contemporary management has shifted toward image-guided percutaneous catheter drainage, which achieves high success even for ruptures, whereas historical surgical series reported substantially higher mortality in free intraperitoneal rupture.^13)^ We found no reports specifically linking peritonitis to cyst puncture or fenestration in the PLD setting; thus, the literature supports rupture as the predominant mechanism. In our patient, postoperative recognition of amebiasis and the subsequent clinical course are best interpreted within this framework.

Emerging evidence suggests that the intracystic environment in PLD is not immunologically neutral but rather characterized by chronic, cytokine-rich inflammation and dysregulated cholangiocyte signaling, including interleukin-6/interleukin-8 and vascular endothelial growth factor pathways.^14,15)^ Conceptually, such a milieu—together with cyst stasis, intermittent hemorrhage, and relative hypoxia—could provide “fertile soil” that facilitates survival and proliferation of *E. histolytica* once trophozoites reach the liver via the portal circulation. In this framework, PLD would function not only as an anatomic substrate but also as a potential intrinsic risk modifier for protozoal infection of cystic spaces. However, our data cannot establish directionality or causation. We did not perform preoperative cyst fluid cytokine profiling, and we cannot discriminate hematogenous parenchymal seeding with secondary cyst involvement from primary intracystic infection. Given the rarity of such presentations and the single-case nature of our report, we regard this mechanism as biologically plausible yet hypothesis-generating. Prospective studies with cyst fluid immunophenotyping and pathogen-specific assays would be needed to test this association.

Elevated serum CA19-9 in patients with large or multiple hepatic cysts may reflect cyst epithelial secretion with intraluminal concentration and subsequent translocation into the bloodstream due to cyst wall stretching, rather than biliary obstruction or malignancy.^16)^ Although cyst-fluid CA19-9 was not measured in our case, the absence of cholestasis and the imaging findings make a cyst-related source the most likely explanation; we acknowledge this as a limitation.

To date, only a few cases of ALA arising in the context of PLD have been reported. Assantachai et al. reported a case initially suspected to be a malignant hepatic lesion, where biopsy and drainage revealed *Klebsiella* species, suggesting a PLA. However, histopathological analysis later identified numerous amebic trophozoites within the cyst wall, leading to a revised diagnosis of ALA, which was successfully treated with ceftriaxone, metronidazole, and surgical drainage.^17)^ Cholongitas described a 93-year-old patient with multiple hepatic cysts presenting with fever and malaise. Imaging findings indicated cyst wall thickening and perilesional edema, suggestive of an abscess. The diagnosis of ALA was confirmed by a positive indirect hemagglutination test for *E. histolytica*. The patient responded well to metronidazole followed by iodoquinol therapy.^18)^ Rana et al. reported an elderly male with PLD and a large hepatic cyst containing yellow fluid. Histopathological examination of the cyst wall confirmed the presence of amebic trophozoites, and serological testing also supported the diagnosis. The patient showed favorable recovery with metronidazole after surgical drainage.^19)^ Taken together, these reports suggest that although ALA in the setting of PLD is extremely rare, it can occur and may be misdiagnosed initially due to atypical clinical and imaging features. In patients with infected hepatic cysts and negative bacterial cultures, amebiasis should be considered as a differential diagnosis. Furthermore, these cases underscore the pivotal role of histopathological analysis in the diagnosis of atypical infections when conventional microbiological tests are inconclusive.

## CONCLUSIONS

For high-risk patients with complex hepatic cysts of uncertain cause, a staged approach is recommended: begin with percutaneous aspiration and targeted fluid analyses—including testing for *E. histolytica*—before considering invasive surgery. This strategy both mitigates operative risk and increases the likelihood of identifying ALA, which should be considered in atypical or culture-negative cases, across cystic liver disease presentations, not only in specific entities.

## DECLARATIONS

### Availability of data and materials

The datasets supporting the conclusions of this article are included within the article.
